# First Forensic Case of Fatal *Salmonella typhimurium* var. Copenhagen Gastroenteritis Diagnosed by Postmortem Microbiology

**DOI:** 10.1097/PAF.0000000000001107

**Published:** 2025-12-10

**Authors:** Anna Laura Santunione, Jessika Camatti, Rossana Cecchi

**Affiliations:** * Department of Biomedical, Metabolic and Neural Sciences, University of Modena and Reggio Emilia, Modena; † Department of Medicine and Surgery, University of Parma, Parma, Italy

**Keywords:** postmortem microbiology, *Salmonella typhimurium* var. Copenhagen, forensic autopsy, forensic pathology, sudden natural death

## Abstract

Postmortem microbiology (PMM) is increasingly recognized as a key tool in forensic medicine, particularly in cases lacking antemortem data or when autopsy and histology are nonspecific. *Salmonella enterica* serovar Typhimurium, including the Copenhagen variant, is a major cause of foodborne disease. While most cases are self-limiting, severe, sometimes fatal outcomes have been reported. A 32-year-old previously healthy man developed profuse vomiting and was found dead the following morning. Autopsy revealed abundant bilious gastric content and liquid stools, without evidence of trauma or intoxication. PMM yielded abundant pure growth of *S. typhimurium* var. Copenhagen from gastric content, while no viral, protozoal, or other bacterial pathogens were detected. Histology revealed cerebral and pulmonary edema and diffuse gastric mucosal inflammation. Toxicology was negative. The integration of autopsy, histology, toxicology, and PMM supported the attribution of death to acute infectious gastroenteritis due to *S. typhimurium* var. Copenhagen. A severe dehydration leading to electrolyte imbalance and fatal cardiac arrhythmia was considered the most plausible mechanism of death. The rapid fatal course was explained by the absence of fluid replacement or medical intervention. This report underscores the value of rigorous PMM protocols not only for cause-of-death determination but also for strengthening infectious disease surveillance.

S*almonella enterica* subsp. *enterica* serovar Typhimurium is one of the most common etiological agents of bacterial gastroenteritis worldwide, transmitted mainly through contaminated food products. Within this serovar, the Copenhagen variant (var. Copenhagen) has been recognized as an epidemiologically important clone. Large outbreaks in the United States during the late 1990s linked to the consumption of unpasteurized dairy products highlighted its capacity to cause widespread disease and underscored its public health relevance.^1,2^

Strains of *S. typhimurium* var. Copenhagen have been isolated from human, animal, and environmental sources, with evidence that pigeons may act as a reservoir capable of transmitting the pathogen to humans.^3^ A hallmark of this variant is its frequent association with multidrug resistance (MDR), including the globally disseminated DT104 clone, which carries resistance to multiple antimicrobial classes and has been described as a truly international epidemic strain.^4–6^ This multidrug-resistant phenotype not only complicates therapeutic management but also increases the risk of severe or prolonged infections.

While most cases of salmonellosis are self-limiting, severe complications have been documented. In particular, *Salmonella* septicemia and myocarditis have occasionally been reported as fatal outcomes, even in previously healthy individuals.^7–9^ These observations emphasize that infection with *S. typhimurium*, including its Copenhagen variant, should not be considered invariably benign. Instead, it represents a pathogen of considerable clinical and forensic interest, with the potential for lethal outcomes in the absence of timely medical treatment.

We present a case of fatal *S. typhimurium* var. Copenhagen gastroenteritis diagnosed primarily through postmortem microbiology.

## CASE REPORT

A 32-year-old previously healthy man developed sudden and profuse vomiting in the evening and was found dead the following morning in his bedroom. Background information was limited, as the decedent was an asylum seeker living in shared accommodation with other individuals who led largely solitary lives. According to his flatmates’ statements, he had vomited repeatedly on the evening before his death. No information on food consumption could be obtained, and therefore, no food testing was possible. In addition, no family medical history was available, as the decedent had no identifiable kin.

The Public Prosecutor ordered a complete forensic autopsy, performed within 48 hours of death and preceded by an on-site inspection. At the scene, investigators documented abundant bilious and alimentary vomitus on the bed and surrounding floor, with no evidence of trauma, drug use, or third-party involvement.

**FIGURE 1:**
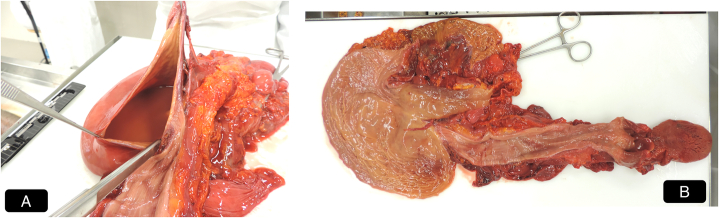
Macroscopic findings. A, Distended stomach filled with bilious and alimentary content, consistent with abundant gastric fluid material. B, Gross appearance of the esophageal and gastric mucosa, without appreciable lesions.

At autopsy, postmortem microbiology (PMM) was carried out in accordance with ESGFOR (ESCMID study group of forensic and postmortem microbiology) recommendations.^10^ Blood was collected from the heart before dissection, by needle aspiration. Gastric content was aspirated with sterile syringes before dissection, after cauterization and disinfection of the gastric serosa to minimize contamination risk. The samples were immediately sealed in sterile containers, labeled under chain of custody, and transported under controlled conditions to the microbiology laboratory. Upon subsequent opening of the gastrointestinal lumen, the tract contained abundant fluid material; the stomach was distended with bilious and alimentary content (Fig. 1A), and the colon showed liquid brown stool without formed fecal matter.

The heart (310 g) was examined, with no evidence of pathologic alterations. The mucosal surfaces of the esophagus and stomach appeared grossly unremarkable, without visible lesions (Fig. 1B). No other significant pathologic findings were observed apart from generalized visceral congestion and pulmonary edema and congestion. Tissue specimens were collected for histologic analysis. Peripheral blood and urine were secured for toxicological analysis.

Microbiological examination was performed by inoculating gastric content onto selective culture media for enteric pathogens. This yielded abundant growth in the pure culture of *S. typhimurium* var. Copenhagen (antigenic formula: O:4 [5−], [27−]; H: i+). Antimicrobial susceptibility testing showed resistance to ampicillin (MIC ≥32 µg/mL), while the isolate was susceptible to all other tested agents, including β-lactam/β-lactamase inhibitor combinations, third-generation cephalosporins, carbapenems, aminoglycosides, and tigecycline. Testing for common enteric viruses (Adenovirus F40/41, Astrovirus, Norovirus, Rotavirus A, and Sapovirus), bacteria (*Salmonella*, *Campylobacter* spp., *Yersinia enterocolitica*, *Vibrio cholerae*, *Plesiomonas shigelloides*, *Escherichia coli*, *Shigella*, and *Clostridium difficile*), and protozoa (*Cryptosporidium* spp., *Cyclospora cayetanensis*, *Entamoeba histolytica*, and *Giardia lamblia*) was performed and resulted negative. Blood cultures were also negative. HIV testing was not performed, as there was no known medical history suggestive of immunodeficiency, and autopsy findings showed no evidence of opportunistic infections or other indicators of immunosuppression.

**FIGURE 2:**
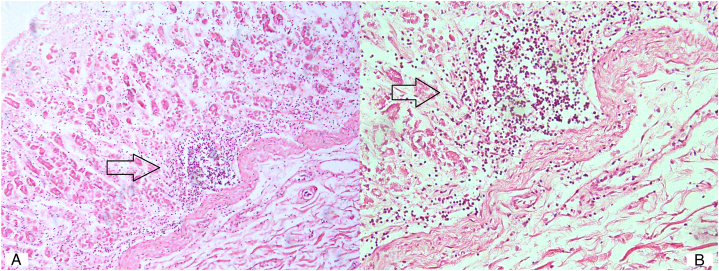
Histology of the stomach showing a diffuse lymphomonocytic infiltrate within the mucosa and lamina propria (arrows). A, ×10, B, ×40.

Toxicological analyses of blood and urine were negative for ethanol, drugs of abuse, and psychoactive medications. Histologic examination revealed cerebral and pulmonary edema. Histologic analysis of cardiac tissue showed no relevant findings, in particular, no evidence of myocarditis. In the stomach, a diffuse lymphomonocytic infiltrate involving the mucosa and lamina propria was observed (Fig. 2), consistent with an acute inflammatory response. No additional relevant findings were detected.

The integration of autopsy, histology, toxicology, and PMM was jointly performed by the forensic pathologist and a microbiologist experienced in PMM. Taken together, these elements supported the diagnosis of fatal acute gastroenteritis due to *S. typhimurium* var. Copenhagen.

A severe dehydration leading to electrolyte imbalance and fatal cardiac arrhythmia was considered the most plausible mechanism of death. Although vitreous fluid was not obtained during the examination and electrolyte testing could not be performed, this hypothesis is consistent with the rapid fatal course in the absence of fluid replacement or medical intervention.

In compliance with Italian law (D.M. 15/12/1990), deaths due to notifiable infectious diseases must be reported to the local Public Health Authority (*Azienda Sanitaria Locale, ASL*). This case was promptly reported as required. Epidemiological investigations were undertaken to identify a possible food source, but unfortunately, no conclusive results were obtained, as the decedent lived a solitary life in a shared accommodation for asylum seekers and no information on his recent meals could be retrieved.

## DISCUSSION

This case illustrates how PMM can provide decisive evidence in cause-of-death attribution,^11,12^ particularly when autopsy and histology yield nonspecific findings. The isolation of *S. typhimurium* var. Copenhagen from gastric content, obtained under controlled conditions and within a short postmortem interval, was consistent with the clinical history of profuse vomiting and the autopsy documentation of liquid intestinal content. The congruence between the anatomic site, the prodromal symptoms, and the absence of competing causes strongly supports the inference of fatal acute gastroenteritis.

The interpretation of microbiological findings in forensic medicine requires careful discrimination between true antemortem infection and postmortem bacterial translocation (PMBT) or contamination. Enteric bacteria may migrate postmortem into blood and sterile compartments, especially with increasing postmortem interval (PMI).^13,14^ In this case, however, the pathogen was isolated from gastric content—a biologically plausible site in light of the clinical history, with pure abundant growth and no polymicrobial contamination. Sampling within 48 hours of death further reduced the likelihood of PMBT. This anatomic congruence markedly strengthens the probative value of the finding.

Several authors have highlighted that PMM results acquire meaning only when interpreted within a broader multidisciplinary framework. Diac et al^15^ demonstrated that microbiological evidence is reliable only when integrated with histology, toxicology, and circumstantial data. Similarly, Tambuzzi et al^16^ reported that combining PMM with histopathology significantly increases diagnostic accuracy and reduces the number of undetermined causes of death. ESGFOR guidelines also recommend standardized sampling, surface disinfection or cauterization, and rapid transport to the laboratory.^10^ All these precautions were strictly followed in the present case, lending robustness to the conclusion.

The Copenhagen variant of *S. typhimurium* has been repeatedly implicated in foodborne outbreaks^1,2^ and is often associated with multidrug resistance, particularly in the DT104 clone.^4–6^ Pasmans et al^3^ also documented its zoonotic potential, with pigeon isolates virulent for humans. In our case, antimicrobial susceptibility testing revealed resistance to ampicillin, while the isolate remained susceptible to β-lactam/β-lactamase inhibitor combinations, third-generation cephalosporins, carbapenems, aminoglycosides, and tigecycline. This profile diverges from the classic MDR phenotype of DT104, underscoring the heterogeneity of resistance among Copenhagen strains. From a forensic perspective, the antibiogram emphasizes the plausibility that survival would likely have been possible had timely treatment been provided, further supporting the attribution of death to untreated infection. A limitation of this case is the absence of molecular genetic testing, which could have provided additional insights into potential genetic predisposition to arrhythmia or infection susceptibility.

In addition to its forensic implications, this case also offers an opportunity to briefly review the epidemiology of this pathogen. *Salmonella typhimurium* var. Copenhagen represents the O:5-negative variant of *S. typhimurium* that has been increasingly isolated from both livestock and avian reservoirs. Originally described as a pigeon-adapted strain responsible for paratyphoid disease in Columba livia, it has now been recovered from cattle, swine, and other domestic animals, indicating a broad zoonotic potential.^17^ Foodborne transmission remains the major route of human infection. Integrated European surveillance data confirm that *S. typhimurium*—including its monophasic and Copenhagen variants—is most frequently associated with contaminated meat and dairy products, particularly undercooked poultry, pork, beef, and unpasteurized milk or cheese.^18^ Direct zoonotic transmission from birds has also been documented: healthy or subclinically infected pigeons may shed *S. typhimurium* var. Copenhagen and disseminate it over long distances during racing flights, occasionally leading to environmental contamination.^19^ Recent molecular studies have demonstrated that *S. typhimurium* var. Copenhagen isolates from domestic pigeons share close genetic relatedness with human clinical strains and harbor major virulence genes such as invA, stn, and spvC, supporting their zoonotic potential.^20^ Moreover, wild and synanthropic birds can act as maintenance reservoirs rather than primary sources of human infection, recycling Salmonella within farm environments and contaminating feed or water through fecal shedding.^21^

Compared with other *S. typhimurium* strains, *S. typhimurium* var. Copenhagen shows host-adaptation traits in avian species—notably phage types DT2 and DT99—and variable antimicrobial resistance patterns.^21^ While the DT104 clone typically displays a multidrug-resistant ACSSuT profile, our isolate showed resistance only to ampicillin,^17,19^ highlighting the genetic heterogeneity within the Copenhagen variant. Collectively, these findings underscore the complex epidemiology of *S. typhimurium* var. Copenhagen, bridging zoonotic, foodborne, and environmental pathways that may converge in sporadic human infections. Although most salmonellosis cases are self-limiting, severe and sometimes fatal outcomes—including septicemia and myocarditis—have been reported even in previously healthy individuals. Simonsen and Falk^22^ described a fatal myocarditis associated with *S. typhimurium*; Burt et al^7^ reported myocarditis secondary to *Salmonella* septicemia, and more recently, Paschalis et al^23^ described myocarditis following *Salmonella* gastroenteritis in an immunocompetent young woman.

These reports, together with the present case, emphasize the forensic value of PMM in detecting unusual or unexpected infectious deaths, ranging from bacterial gastroenteritis to viral myocarditis and fulminant septicemia.

Beyond the diagnostic dimension, this case emphasizes the forensic importance of methodological rigor in PMM. Nodari et al^24^ highlighted that PMM not only improves cause-of-death certification but may also contribute to epidemiological surveillance and outbreak detection. Ventura Spagnolo et al^25^ also underscored the broad applications of forensic microbiology. Nonetheless, significant limitations remain: systematic reviews^13,14^ warn that microbiome-based PMI estimation is still hindered by methodological heterogeneity. Looking forward, advances in next-generation sequencing (NGS) and artificial intelligence may expand the utility of PMM. As Hu et al^26^ suggest, combining microbial signatures with multimodal data sets could improve discrimination between contamination, colonization, and true infection. Integrating these technologies with classic culture and histopathology may represent the next step toward standardizing PMM in forensic practice, in line with other postmortem applications of molecular and immunohistochemical markers.^27–29^

From a forensic perspective, this case highlights several key considerations.

Fatal infectious deaths diagnosed primarily through PMM are uncommon and present distinctive diagnostic and interpretive challenges, which can be addressed through standardized protocols and interdisciplinary collaboration in forensic sciences. To our knowledge, no previous forensic report has described a fatal outcome due to *S. typhimurium* var. Copenhagen identified through PMM. This case demonstrates that postmortem detection of *S. typhimurium* var. Copenhagen in the gastric content—supported by the clinical history, autopsy findings, histology, toxicology, and antimicrobial susceptibility results—allows a forensic-level attribution of death to acute infectious gastroenteritis. In the present case, social vulnerability and limited access to health care likely contributed to the fatal outcome, as the subject was apparently unable to recognize his deteriorating condition or seek emergency medical assistance.

Moreover, fatal infections may have not only forensic relevance but also important implications for public health surveillance and outbreak prevention. Beyond cause-of-death attribution, PMM may therefore serve as a bridge between forensic pathology and public health, facilitating the notification of infectious diseases of public concern and supporting the timely initiation of epidemiological investigations.
